# Some Needed Legislation

**Published:** 1897-08

**Authors:** B. W. Bizzell

**Affiliations:** Atlanta


### SOME NEEDED LEGISLATION.

ATLANTA, July 20, 1897.

*Atlanta Medical and Surgical Journal:*

At the last meeting of the Georgia State Medical Association, held in Macon, I introduced a resolution providing for the appointment of a legislative committee to frame and present to the next legislature certain bills tending to regulate and elevate the practice of medicine within the State. For the benefit of those who were not present at the last meeting, I take this means of presenting the resolution as offered, and also give the committee appointed by the president, Dr. Morgan:

1. *Resolved,* That a Central Committee of nine members of this Association be appointed by the chair, whose term of office shall be as follows: Three for one year, three for two years, and three for three years, appointing three new members each year to fill expired terms of office. Whose duty it shall be to frame and present to the next legislature the most feasible plan for the introduction in court of medical expert testimony in criminal cases.

2. To report and prosecute all violations of the State law governing the practice of medicine.

3. The Central Committee to organize sub-committees in each county whose duty it shall be to assist in the framing and passage of a bill, and report all irregularities and violations of existing medical laws in their respective counties.

4. It shall be the duty of said Central Committee to meet in Atlanta during the meeting of the next legislature, or such other time as is deemed best, to present and assist in the passage of said bill.

5. That these sub-committees, the Central Committee concurring, are hereby empowered and directed to construct, frame, and adopt such measures as may seem best suited.

6. That this Central Committee be subservient to the State Medical Association and shall report to that body each year in annual session.

Amendment to resolution offered by Dr. Kime, of Atlanta:

1. *Resolved,* That the above committee provided for by Dr. Bizzell's resolution shall also frame and present to the Medical Association at its next meeting the most feasible plan for the prevention of the spread of tuberculosis.

2. Also to report upon the most feasible plan for preventing the law now providing for a State Board of Medical Examiners being repealed, reporting fully to the State Association at the next annual session any and all points affecting this bill, and the workings of said Board of Examiners.

Dr. B. W. Bizzell, Chairman, Atlanta, one year.
Dr. R. B. Barron, Macon, one year.
Dr. Eugene Foster, Augusta, one year.
Dr. E. R. Anthony, Griffin, two years.
Dr. Willis Westmoreland, Atlanta, two years.
Dr. I. H. Goss, Athens, two years.
Dr. E. H. Richardson, Atlanta, three years.
Dr. F. M. Ridley, LaGrange, three years.
Dr. George H. Noble, Atlanta, three years.

As chairman much of the work of this committee will fall on me, and I can hope to accomplish but little without the aid of the profession throughout the State. Every member of the Association and every physician not a member should give any measure tending to elevate the profession his moral support, and if this is done I feel confident the legislature will grant our request and pass the bills as presented.

Perhaps no branch of science had advanced more rapidly than the science of medicine and surgery, which is largely due to the manner of medical education as taught in our schools and to wise legislation in most States forcing a certain element to either move on or perfect themselves for their positions and professions. The first step towards advanced medical teaching was the adoption of a three years' course, instead of two, then the requirements in many schools of certain literary qualifications before the study of medicine is begun. A few years ago the medical schools met and adopted the three years' course, with preliminary literary qualifications, etc. All the medical schools of the South entered this compact except one, and this one subsequently adopted the three years' course by reason of State legislation. Soon after the adoption of the three years' course a law was passed creating a State Board of Medical Examiners, who stand to-day as a safeguard between the laity and the plausible quack who would come to our State. Since the enactment of this law Georgia has ceased to be a dumping-ground for the cast-offs and refuse of other States.

It is now time for more legislation and the creation of other laws equally as important and effective as the one providing for the State examining board.

First, the law specifying the duties of this examining board should be so modified as to require them to examine into the literary attainments of applicants before going into the medical examination. The same board of medical examiners are eminently qualified for this work, and with but little loss of time give both examinations when necessary.

Second, we need a law to provide for the introduction of medical expert testimony in criminal court cases. For the necessities of such a law I would refer you to the very able paper presented by Dr. J. C. Olmsted and read before the Association at Macon.* It is impossible for me to go into detail here or outline the proposed bills relative to expert testimony or the duties of the State Examining Board, and I simply make the announcement of the Legislative Committee and call on every physician to give us his support and make it his duty to call on his representative and urge him to vote for the bills when presented. I hope to be able to furnish a copy of proposed bills in the next issue of this Journal, and I most earnestly beg of all who are inclined (if any exist) to criticize, to be patient and wait till the bills are completed. B. W. BIZZELL, M.D.

---

One senator at least has announced where he stands in reference to the obnoxious bill to prohibit vivisection in the District of Columbia. In a letter to a committee of the Richmond Academy of Medicine and Surgery, Senator Thos. S. Martin, of Virginia, says in part *(Medical Register)*: "I beg to assure you, and through you to assure your professional brethren in the city of Richmond, that I concur fully in the views you express in reference to this bill. I will use every effort in my power to prevent its passage. There is no danger whatever of its passing at the present special session. There will, of course, be a strong effort made next winter to pass it. I will keep close watch on the matter, and if its passage can be prevented by me you may rest assured it will not pass."

*See June issue of this Journal.

